# Challenges to effective governance in a low income healthcare system: a qualitative study of stakeholder perceptions in Malawi

**DOI:** 10.1186/s12913-020-06002-x

**Published:** 2020-12-14

**Authors:** Sarah C. Masefield, Alan Msosa, Jean Grugel

**Affiliations:** 1 Interdisciplinary Global Development Centre, University of York, York, YO10 5DD UK; 2 Department of Politics, University of York, York, YO10 5DD UK

**Keywords:** Healthcare governance, Malawi, Stakeholders, Universal health coverage, Social accountability, Health care delivery, SDG 3, Low income countries

## Abstract

**Background:**

All countries face challenging decisions about healthcare coverage. Malawi has committed to achieving Universal Health Coverage (UHC) by 2030, the timeframe set out by the Sustainable Development Goals (SDGs). As in other low income countries, scarce resources stand in the way of more equitable health access and quality in Malawi. Its health sector is highly dependent on donor contributions, and recent poor governance of government-funded healthcare saw donors withdraw funding, limiting services and resources. The 2017 National Health Plan II and accompanying Health Strategic Plan II identify the importance of improved governance and strategies to achieve more effective cooperation with stakeholders. This study explores health sector stakeholders’ perceptions of the challenges to improving governance in Malawi’s national health system within the post-2017 context of government attempts to articulate a way forward.

**Methods:**

A qualitative study design was used. Interviews were conducted with 22 representatives of major international and faith-based non-government organisations, civil society organisations, local government and government-funded organisations, and governance bodies operating in Malawi. Open questions were asked about experiences and perceptions of the functioning of the health system and healthcare decision-making. Content relating to healthcare governance was identified in the transcripts and field notes and analysed using inductive content analysis.

**Results:**

Stakeholders view governance challenges as a significant barrier to achieving a more effective and equitable health system. Three categories were identified: accountability (enforceability; answerability; stakeholder-led initiatives); health resource management (healthcare financing; drug supply); influence in decision-making (unequal power; stakeholder engagement).

**Conclusions:**

Health sector stakeholders see serious political, structural, and financial challenges to improving governance in the national health system in Malawi which will impact the government’s goal of achieving UHC by 2030. Stakeholders identify the need for improved oversight, implementation, service delivery and social accountability of government-funded service providers to communities. Eighteen months after the introduction of the policy documents, they see little evidence of improved governance and have little or no confidence in the government’s ability to deliver UHC. The difficulties stakeholders perceive in relation to building equitable and effective healthcare governance in Malawi have relevance for other resource-limited countries which have also committed to the goal of UHC.

**Supplementary Information:**

The online version contains supplementary material available at 10.1186/s12913-020-06002-x.

## Background

Low-income countries face many essential healthcare challenges, ranging from how to prioritise limited resources to patchy service provision (both in terms of the interventions available and geographical coverage) [1, 2]. In 2015, 193 United Nations (UN) member states, including low income countries, became signatories of the Sustainable Development Goals (SDGs) [3] and committed to the goal of Universal Health Coverage (UHC) by 2030. Healthcare, and delivery of the SDGs more widely, are underfinanced in low income countries, significantly affecting service delivery, and making it highly unlikely that the SDGs and UHC global targets will be achieved by 2030 [1, 4–6]. Long-term challenges in the health system can be understood through the lens of health sector governance, not least because effective service delivery depends on good healthcare governance [6, 7]. Governance, broadly, refers to the concept of institutional quality – the World Bank specifies that good governance requires stakeholder voice and accountability, political stability, government effectiveness, regulatory quality, rule of law and control of corruption [8]. Yet, although issues of service delivery in low income countries are well documented, less attention has been paid to healthcare governance and its vital importance in managing resources in resource-constrained environments.

Good governance in the health sector refers to the making of pro-health legislation and frameworks for the implementation of strategic policies combined with effective regulation, monitoring, system design and social accountability [9]. According to the World Health Organization, good governance of health requires: maintenance of the strategic direction of policy development and implementation; monitoring the health system to detect adverse trends in efficiency; advocating for health in national development; regulating the behaviour of health stakeholders, (including financers and healthcare service providers); and establishing effective and transparent social accountability mechanisms. These are difficult to deliver in situations where resources, capacity, staffing and infrastructure remain limited in practice and the health system (financing and services) is often distributed (e.g. between the government, donors, non-government organisations (NGOs) and faith-based providers). For this reason, an essential route to improving the governance of the health system in low income settings is through working effectively with non-government stakeholders [10, 11].

Health stakeholders can be defined as organisations and individuals involved in the production, consumption, management, regulation or evaluation of a specific health activity, including governance of the health system or health policy development [12]. Eliciting stakeholder perspectives allows healthcare to be seen from multiple angles, enabling exploration of differences and similarities in the understanding of specific issues (e.g. health services or policies) and perceived health needs of different individual stakeholders or groups (e.g. policy-makers versus service users) [12, 13]. This research can be used to influence the development or refinement of new policies, services and governance processes, including those focused on delivering UHC [13, 14].

The coronavirus pandemic has highlighted the challenges of health information monitoring and inequalities of access in low income countries with weak health systems and without UHC [15, 16]. In both the developing and developed world, there are now citizen calls both for more urgent responses to the pandemic, for UHC and accountability of governments for their responses [17, 18]. It is therefore particularly relevant at this time to explore the tensions between governments and stakeholders in relation to the capacity of the health system to respond to pressing health needs (e.g. financing, workforce capacity, resource distribution, access to medicines) as limitations in these areas critically affect capacity to respond to major health crises, whilst failures to do so can further undermine and weaken health systems [19–21].

We use Malawi, a UN member, as a case study for investigating perceived challenges in healthcare governance and health system functioning in low income countries moving towards UHC.

### Malawi context

Malawi is one of the poorest countries in the world [22]. It has low per capita spending on health of 39.2 USD, which is significantly lower than the Sub-Saharan Africa average of 98 USD [23]. The Government of the Republic of Malawi has signed the Abuja Declaration to commit at least 15% of the national budget to health [24], but only allocated 9.8% in 2018. As a low-income country with limited tax revenue, a higher health allocation would still deliver an under-funded health system [25]. For example, for the period 2012/13–2014/15, the government only accounted for an average 25.5% of the total health expenditure (and households for 12.9% i.e. direct payment for health services) [24]. However, it is worth noting that health gains can be achieved with limited resources - Malawi was one of few countries to meet the Millennium Development Goal for child health [26].

In 2017, the government produced the National Health Policy II (NHP II), which is closely aligned with SDG 3 (ensure healthy lives and wellbeing for all at all ages) [27]. In this, UHC is defined as ‘a situation where all people have access to quality essential healthcare services and essential medicines and vaccines without suffering undue financial hardship as a result of accessing care’. The policy specifies the following objectives, to be met between 2017 and 2030, to strengthen the health system and achieve UHC:

1. Improve service delivery by ensuring UHC of essential health care services, paying particular attention to vulnerable populations.
2. Provide effective leadership and management that is accountable and transparent at national, and local authority levels.
3. Increase health financing equitably and efficiently and enhance its predictability and sustainability.
4. Improve availability of competent and motivated human resources for health for effective, efficient, quality and equitable health service delivery.
5. Improve the availability, accessibility and quality of health infrastructure, medical equipment, medicines and medical supplies at all levels of healthcare.
6. Reduce risk factors to health and address social determinants of health and health inequalities.
7. Strengthen capacity in health research and health information system management for evidence-based policy-making.

However, concerns have been raised (e.g. in academic and media outlets in 2019) about the predictability and sustainability of the health system, especially in its ability to finance and achieve its health objectives, including UHC [28–30]. For example, significant improvements in service delivery cannot be made whilst the staff vacancy rate for healthcare facilities remains very high (50%) and some communities are up to 35 km away from their nearest facility [29, 30].

Healthcare delivery is mainly via government facilities (63%), which have some service limitations but are free at the point of access. Healthcare is also delivered by the Christian Health Association of Malawi (CHAM; 26%) for a small user fee, and by private for-profit and civil society providers (11%) [28]. The health system is highly dependent on donors. In 2014/15 donor aid contributed 53.5% of the nation’s total health expenditure. However, this was down from 68.3% in 2012/13 due to donors withdrawing direct financing (via a basket fund) for the Ministry of Health’s (MoH) strategic and implementation plans in response to a financial corruption scandal that broke in 2013, known as Cashgate [28]. This erosion of donor confidence produced an accountability crisis across the health sector. The financial arrangements and trust between civil society organisations (CSOs) and donors were also adversely affected, as donors feared widespread government corruption within the government and non-government health system.

The government recognises the essential role of governance in enforcing and monitoring the actions required to achieve their health objectives, and leadership and governance were identified as priority areas in the Health Sector Strategic Plan II 2017–2022 (HSSP II) [24]. This is the strategic framework for the NHP II which focuses on strengthening governance in the health sector to improve efficiency and optimise existing resources (human, financial, material), particularly by improving the domestic financing mechanisms. The Minster for Health acknowledges that the country’s health sector is highly dependent on external financing, and the vital importance of continued aid to support health gains. Demonstrating improved governance, including building better relationships with stakeholders, is essential for rebuilding the damaged relationship between the government and donors in order to achieve continued donor contributions and a more coordinated approach to the funding and provision of healthcare in Malawi [10, 24].

There have been, consequently, a series of measures to improve the governance of the health sector. For example, in 2018 the MoH created the new role of hospital ombudsman to ensure better service delivery in public and CHAM health facilities with greater social accountability between the facilities and communities via improved connections between the service users and providers [31]. However, significant concerns regarding health sector governance, particularly around financial and resource efficiencies, and tensions between government stakeholders remain [32], some of which we explore in this paper. Given the commitment of the government to improve healthcare governance, outlined in the NHP II and HSSP II, identifying these challenges is critical, yet, to our knowledge, no previous research has investigated the concerns of people operating within the health sector in Malawi and the issues they perceive as the challenges to better governance and thus to greater health system efficiency. Further, none has reflected upon the synergies between Malawian health policy and healthcare governance in practice.

## Methods

We reported our research using the Consolidated criteria for Reporting Qualitative research (COREQ) checklist [33]. Our study has an exploratory qualitative study design, with largely unstructured interviews conducted with a range of health stakeholders. We opted to speak to stakeholders individually, rather than through focus groups, due to the potentially sensitive nature of the topic when many stakeholders work closely with the government. This allowed us to be certain that when stakeholders repeated concerns, they were expressing their own opinions and not being led by the reflections of others.

### Participants

Using a purposive sampling strategy, health stakeholders working in decision-making or regulatory roles in the government-funded health system or who have advocated for change in the health sector at the government-level were identified via a mapping exercise and invited to interview. Using their local and specialist knowledge from conducting health research in low income countries, and in Malawi specifically, AM and Thanzi la Onse project partners in the College of Medicine at the University of Malawi and the Oversees Development Institute identified government and non-government institutions providing health services or performing healthcare governance activities and located in Malawi [34, 35]. This was supplemented by electronic searches of health sector websites and registries (such as the register of the Council of Non-Governmental Organisations and delegate lists from health conferences and workshops) and searching health-related newspaper articles in The Nation and The Times. Key individuals (*n* = 22) within these institutions and located in Lilongwe and Blantyre were identified. These urban locations were selected for logistical reasons and due to limited resources.

We performed 22 face-to-face interviews in a private space in the participants’ place of work, the preferred location of all participants. Everyone approached for interview consented to participate. The sample consisted of representatives from the organisation types: international NGOs (*n* = 3); faith-based NGOs (*n* = 2); CSOs (*n* = 9); local government and government-funded organisations (*n* = 6); and governance bodies (*n* = 2). We sought a range of rural and urban, government, community, and citizen level perspectives. We included central and district level representatives as, due to decentralisation, the Blantyre and Lilongwe District Health Offices are not part of central government or the referral health system. Including the district level also provided perspectives of healthcare delivery in rural settings, as the districts include significant rural (and socioeconomically deprived) populations. The CSO and governance bodies engaged directly with citizens, community groups and civil rights movements/projects.

### Data collection

Ethical approval for the study was received from both the University of York (6 July 2018) and from the College of Medicine in the University of Malawi (16 October 2018). The interviews were conducted by AM in a combination of English and Chichewa between December 2018 and February 2019.

As the research was exploratory, open questions were asked in order to capture each person/organisation’s experience and perception of the functioning of the health system and healthcare decision-making in Malawi. Guiding questions were set by AM and JG and reviewed by the ethics committees. For example, describe the role of your department/institution in the resource allocation process; what challenges do you see in Malawi’s health sector? A semi-structured interview schedule (Supplementary material 1) was used to enable the eliciting of further detail on topics of importance to the interviewee, including governance (which was raised to some extent by every interviewee). Accordingly, and given the diverse stakeholders, the questions varied in each interview.

Each interviewee was informed as to the purpose of the research and given an information sheet before giving signed consent to participate in the interview study. Six consented to audio recording via dictaphone. The remainder (*n* = 16; 72.3%) consented to field notes with the possibility of citing direct quotations but not audio or video recording as they wanted to speak freely and without concern for government (or employer/colleague) reprisal. Guidelines for making field notes and their integration with study data were followed [36]. The verbatim transcribed recordings and field notes (with direct quotations) for the non-recorded interviews comprise the transcripts used in the analysis. Due to the potentially politically sensitive nature of the content, every effort has been made to anonymise the individual participants in the reporting of this research. Attribution is made by type of organisation and participant number only.

The interviews lasted between 45 min and 1.5 h.

### Data analysis

Where necessary, the transcripts were translated into English by AM. Once all the interviews had been conducted, the analysis was performed by SM using inductive thematic analysis, whereby the coded categories were derived directly from the data (rather than the data being coded to support a pre-existing theory) [37, 38]. This is an iterative process of abstraction where units of the data (words, sentences and paragraphs from the interview transcripts and field notes) relating to the broad topic of healthcare governance were identified and combined/grouped with similar content to form major categories and subcategories [39, 40]. This was an appropriate approach for an exploratory study on a topic with no known studies [37].

The exploratory study design and analysis were expected to produce a list of perceived governance issues, grouped into categories. Given the breadth of the data expected, we did not seek during the interviews or the analysis to identify the extent to which different interviewees or organisation types agreed on specific potential governance issues. For this reason, a data saturation approach was not used in the analysis. Governance issues raised by one or more people are reported. Where available, direct quotations are used to illustrate interviewee perceptions. Elsewhere, impressions and scenarios described by the interviewees and recorded in the field notes are presented and attributed with participant numbers.

The analysis was performed between November 2019 and January 2020 in Nvivo 12 software.

## Results

Although there was some overlap, three major categories (seven subcategories) of perceived governance issues were identified: accountability (enforceability; answerability; stakeholder-led initiatives); health resource management (healthcare financing; drug supply); and influence in decision-making (unequal power; stakeholder engagement).

### Accountability

Stakeholders identified limitations in the accountability of the government to health stakeholders in both the spheres of enforcement (of health decisions made) and answerability (for the impact of these decisions). Challenges focused on the inadequate implementation of health policy and insufficient answerability mechanisms. These included absent implementation plans, poor dissemination of national policy to other levels of healthcare (regional, city, service), limited processes to provide a feedback mechanism between service providers and the government and citizen review in government health policy and governance structures. In response to the perceived inadequacies of government-led healthcare governance, initiatives led by service-providers, NGOs and CSOs have emerged to hold the government to account for their health policy decisions and poor resource management.

#### Enforceability

An interviewee from a healthcare governance body stated ‘there is always a big difference between the rosy policies or strategies and implementation. Implementation leaves a lot to be desired’ (Participant(P)21). The CSOs shared this view (P6, P9, P11, P13), with one identifying a nationwide need for more affordable, available and accessible health services, but ‘these issues are not very clear in the strategic plan and policy’ (P6). It was considered highly unlikely that improvements would be made without a policy-level commitment to making advances in these areas combined with a clearly defined (monitored and enforced) implementation plan: ‘beyond development of strategies and policies, have they thought about resolving the current recurring problems? (P9)’. This interviewee also referred to the need for greater governance of the drug supply chain and human resources (discussed under ‘health resource management’).

A specific example of poor governance was the Charter on Patients’ and Health Service Providers’ Rights and Responsibilities which was developed by the MoH together with CSOs but was never implemented (P13). Thus, there is a government commitment to the protection of human rights in the service delivery context, but little public awareness of these rights and they are not protected in practice. Others found the implementation of policy to be ad hoc. For example, there have been sporadic visits by District Health Officers to CHAM facilities to monitor compliance with government standards in accordance with the service level agreements (SLAs) (P5).

The partial implementation of the government policy of decentralisation was viewed as a cause of poor governance in the health sector (P15, P19, P20). The move to devolve power from the government to local level authorities (district and city) had resulted in greater budgetary constraints at these levels with limited dissemination of government policy via implementation guidelines. The city assemblies were not mentioned in the health policies or HSSP II and had not been involved in developing or reviewing them. The representative felt this was short-sighted of the government as implementation of the Essential Health Package (EHP; the package of essential services identified by the government as the starting point for the move towards UHC), ‘cannot be successful without the active input and involvement of the city assemblies’ as the cities are ‘hosting a significant portion of the population’ (P15). Further, the local authorities felt unable to implement the government policy: ‘in reality, each district orders drugs based on the local needs and dynamics. The EHP in the HSSP II provides an ideal scenario, but in practice we have to respond to local realities’ (P19).

Concerns were also raised from within a government governance body, where it was felt that the knowledge, skills and motivation of the members of these bodies/committees could determine the extent to which the government is held to account: ‘the functioning of the committee relies on expertise of the members [ …] we have to be proactive to facilitate the changes that the country needs’ (P21). When the Chair or membership changes, the body could, therefore, become less effective. The lack of routine and comprehensive communication between the different governance bodies was also mentioned as a weakness:

‘[we have] not previously engaged with the parliamentary committee on health, although they are an important player in the accountability for health. However, they have interacted only at the launch of the NHP II, which cannot be considered as formal or in depth engagement’ (P22).

One instance where the communication procedure has been formalised (and is reported as greatly improved as a result) is between the Office of the Ombudsmen and the hospital ombudsmen (P22).

#### Answerability

Efforts to provide answerability mechanisms and their limitations were discussed. These included processes to provide a feedback link between service providers and the government and the extent of citizen review in government health policy and governance structures.

Frustration was expressed about the lack of citizen-level awareness and advocacy for greater government social accountability (P21, P8). A representative of a government-funded governance body stated that the process of parliamentary committee reviews is responsive, whereby issues are brought to their attention, triggering a review. However, they found that ‘Malawians are not proactive in demanding the committee’s legislative intervention’, giving the example of the Mental Health Act, which they said was out of date, yet no one has requested a review or amendment (P21).

NGO and CSO representatives remarked on a sense of apathy towards governance among the general population (P2, P8). A Malawian representative of an international NGO stated:

‘the problem with most Malawians is that they view human rights as a charity or a favour, not as an entitlement. When government fails to uphold or protect their rights, they therefore are not to demand rights as an entitlement that they ought to have. That way, issues like poor service delivery continue without any consequences’ (P2).

They criticised the government and courts for not clarifying the ‘state obligations in the protection of the right to health’ and the lack of discussion on the impact of economic, social and cultural rights on health (P2).

Conversely, it was also felt that when NGOs and CSO representatives did try to increase government recognition of specific health needs, the government response was tokenistic - it did not result in sustainable changes to health policy or effective implementation. For example, in response to civil society advocacy efforts, drugs for a vulnerable patient group were purchased by the government via the parliamentary committee for health. However, ‘it was a once off reactive purchase’, the supply was insufficient for the demand, and there was no distribution plan so the drugs could only be accessed via two hospitals in Lilongwe (P3). There was apparently no effective mechanism by which the group could seek an independent review of the situation.

There was, however, some evidence of government efforts to roll out accountability measures reportedly backed by the public. The Office of the Ombudsman had ‘shifted its focus towards service delivery by systematically reviewing institutional processes to recommend corrective measures’ (P22). Interns, being trained as hospital ombudsmen, had been placed in all four central (government-run) hospitals after ‘positive media reporting resulted in public and institutional demands for the idea to be spread to the central hospitals’ (P22). The representative of the governance body who gave this example also remarked that by recruiting and training up interns specifically for the role, they would not be current or previous MoH staff, thus implying a degree of impartiality. However, a structural issue remained as the ombudsmen report back (and are junior to) the District Health Officer, who has decision-making authority in district administration, ‘it is therefore very difficult for the subordinates to play watchdog over their seniors, even more difficult to sanction or report them for any wrongdoing’ (P22).

There was further evidence of collaboration between the government and other health stakeholders in MoH governance processes. For example, an NGO provided technical support to the government by placing local and international technical experts in the MoH as advisors (but the initiative ended in 2016 when the funding expired) (P2). Another NGO sits on the MoH’s Community Health Technical Working Group (TWG) to contribute expertise on how to build an effective community health system (although they approached the MoH to request to be on the group rather than being invited to participate) (P1). The Malawi Health Equity Network (MHEN) is routinely consulted by the MoH, attending regular and ad hoc meetings, and participating in TWGs (P11). However, not all CSOs are members or feel represented by MHEN:

‘the challenge with MoH’s engagement with NGO stakeholders is that they assume that MHEN is the representative of all health NGOs, but not all NGOs doing work in the area of health are members of MHEN. The organisation’s view is that MHEN can’t replace grassroots voices in the engagement with the MoH. MHEN does not have capacity to represent all voices, simply impossible’ (P13).

#### Stakeholder-led initiatives

In the absence of adequate social accountability, examples were given of how service providers, NGOs and CSOs sought to hold the government to account for limitations in the health system. For example, in response to poor government-led governance of healthcare facilities and service inefficiencies, service providers had introduced structures to increase accountability at the service-level and in their interactions with the MoH (P4, P5, P17, P22). Queen Elizabeth Central Hospital sought greater autonomy from the government by establishing their own information management system and is seeking registration as a public trust. To this end, an independent consultant has been engaged to assess the readiness and capacity of the hospital, and they have prepared trust deeds, a constitution, and Terms of Reference for the board (required documents for registration of public trusts with the Registrar General) (P17).

Another approach was to include MoH representatives on the key management boards of the service providers, thus facilitating direct and ongoing communication with the MoH via a designated person. By including a MoH representative on CHAM’s boards, the ongoing issues of funding deficits and drug stockouts (when required drugs are not available at healthcare facilities) would be fed back to the government, and hopefully addressed, more promptly (P5). They successfully lobbied the government to establish a joint SLA Unit whose sole purpose is to communicate with the facilities, monitor the contracts, pay facility staff directly and respond to any issues (P5). Part of the Unit’s role is to visit the healthcare facilities to review the challenges. Further, CHAM now insist on Memorandums of Understanding with the government to ensure that each of their facilities is the only government-registered and recognised facility in that area (P5). This protects them from the government stipulation that funding can be withdrawn if healthcare facilities are within a 5 km distance of each other.

Efforts were also being made by NGOs and CSOs to increase the social accountability of the government to the public through advocacy and monitoring activities and training (P3, P8, P12). Direct action included pressuring the MoH to respond to health-related incidents, such as aggravated attacks on people with specific health conditions (e.g. albinism) (P3) and for the inclusion of ‘neglected health issues in the national health responses’ (P13). This had resulted in the introduction of a new MoH TWG which includes consideration of these health issues alongside those already receiving a significant focus (e.g. HIV/AIDS) (P13). Other advocacy initiatives were targeted at the national and local government levels and service providers (e.g. hospitals). These included calling on the government to increase the health budget to the level expected under the Abuja Declaration (P9, P11) and educating the public in the need for greater social accountability, thus creating a demand (P8). It was felt that the courts should have (but had not) taken an active role in clarifying the ‘state obligations in the protection of the right to health’ (P2). There were efforts to highlight this and to raise awareness among the public that they can use the court system to demand health services (P8).

Another approach used was to demonstrate to the community the value of specific services with time-limited funding to mobilise them to become accountable for these services and demand the government provide ongoing access (P12). Training initiatives aimed to equip communities and individuals in local government and health service delivery (e.g. health advisory and health centre management committees, faith-based NGO and district health management teams, local government councillors) with skills in budget analysis and monitoring to become advocates for governance and hold to account the service providers that they interact with (P8, P11). For example, manuals have been developed to train service providers on upholding the human rights of vulnerable groups during service delivery (e.g. women, children and sexual minorities) (P8).

Collaboration with other organisations was perceived as strengthening the advocacy efforts of CSOs, such as calling for greater governance to prevent corruption in the health sector. For example, one group found that membership of an international NGO network gave them support with developing a strategic plan to advocate for the creation and adoption of a national response plan in a neglected disease area (P13). Other groups formed a coalition to become the patient voice in government consultation exercises (P8, P11) or lobby international NGOs and donors to advocate for their interests (e.g. drug safety and more health professionals working in their disease area) as they are believed to have more influence with the government and can raise awareness at the international level (P12). These collaborations are also conducting independent monitoring of the national health system and looking at how the government is meeting its legal and policy obligations to publicly hold the government to account for its actions and interventions (or lack of them). For example, monitoring progress towards achieving the UHC target (P8), monitoring the extent to which health interventions specified in the MoH’s policies are being implemented (P11), and assessing whether the health budget approved by Parliament is being implemented (as there is a known deficit in the amount spent versus the amount allocated) (P8, P11, P18). Periodic service delivery satisfaction surveys are also conducted to hold government-funded service providers to account for the quality of the care they deliver (P11).

### Health resource management

Most of the challenges to the governance of health resources arose from underfunding (whether due to the insufficient allocation of funds or corruption) and a lack of interconnectivity (communication and resource distribution) between the different levels of the health system. Specific instances of poor governance were identified for drug supply, exacerbated by the fragmented health system.

#### Healthcare financing

It was acknowledged by several interviewees that a lack of allocated and available (due to low domestic revenue) funding hindered improvements in the health system in general, and governance specifically (P22). The city assemblies do not receive funding from government as they receive locally generated revenue (city or property rates, licences, and service charges). This produces insufficient funds to meet the health needs that the city assembly are mandated to provide: 1) public cleansing services (e.g. waste management, cemetery services); and 2) preventative community health services (e.g. family planning clinics, health education, pest and infection control, HIV prevention and treatment) (P15, P20). Further, the lack of government funding received by the City Assemblies was flawed as it ‘overlooks the reality of disease or health burdens’. The example provided was cholera outbreaks, which typically spread from rural Lilongwe to more urban areas, ‘yet the City Assembly is expected to bear the cost when the crisis hits the urban population’ (P20). It was felt that the City Assemblies should receive some funds from the Ministry of Local Government as the ‘urban populations also pay for other taxes and must get returns through the national budget’ (P20).

When the District and City Assemblies are located in the same city (as in Lilongwe and Blantyre) it was argued that:

‘the District Health Office should be doing more to fill in the gaps [in the City Assemblies’ funding/service provision]. But bearing in mind the financial limitation of the District Assemblies and the District Health Offices, that is a consideration for the City Assembly letting them get away with not covering all the gaps’ (P15).

Accordingly, the health policy cannot be enforced because the City and District Assemblies cannot be made to provide services which they cannot fund. This leads to a mutual acceptance of substandard service delivery at multiple levels. Furthermore, local government representatives expressed frustration at not receiving clearer guidance about resource allocation from the MoH. The government determined the structure for decentralised health resource allocation - health resources are to be decided at the district level by the District Health Allocation Committee - but did not issue any guidance on the composition of the committee or its role in health resource allocation at the regional level (P19). The inference being that it was inefficient for each district to develop their own guidelines and results in inadequate committees.

Issues were also identified between the government and service providers. The government has SLAs with CHAM, paying them to provide healthcare facilities, hospitals and training of healthcare professionals. However, the government frequently fails to meet their contractual obligations:

‘through CHAM we signed what we call them service level agreements, but are they fully adhered to fully? Not really. But we still have to keep on like providing the services because we are also responsible to the communities that we are in’ (P2).

On some occasions, the MoH has delayed payment to CHAM by 4 months (accumulated non-payment without warning/agreement) (P5). In some facilities this has resulted in a poorer quality of service delivery as the staff and essential running costs cannot be paid and drugs purchased (P5):

‘the government is currently failing to honour agreements by defaulting on payments to some of the facilities and as a result, citizens whose only option is to go to CHAM facilities to access health services are being turned back because the hospitals won’t offer free services until the government has paid’ (P8).

Conversely, despite the MoH’s funding constraints, it was reported that one government-funded organisation was able to renegotiate additional funding after the budget ceiling for the year had been set. They received an extra 1 million US dollars by arguing that the allocated budget has a serious shortfall and ‘downscaling would have resulted in public dissatisfaction and diminishing of public support’ (P18). Thus, top-up funding may be possible for organisations who know how to approach and argue their case with the government.

Three interviewees perceived corruption as the greatest ongoing challenge to an effective health system (P2, P8, P9). For example, ‘the problem with our health system may not be about insufficient resources’, inferring that theft and corruption are responsible for the resourcing issues (P9). There was criticism of the government for seeking to introduce user fees to secure additional revenue for the health system, but which would not address the underlying issue of poor resource management:

‘Failure of Malawi’s health system is about corruption because the financial systems are porous. Once we address that problem, then we can talk about whether people can pay user fees. There are many ways to increase availability of resources by avoiding wastage. Malawian people are poor and additional payments won’t address the problem of system failures or drug stockouts. Drug stockouts are a symptom of a dysfunctional system than insufficiency of funding. Even if the idea of user fees was accepted and implemented, there has to be someone to collect the pay and manage the funds. In the current situation, the funds would create more problems than solutions’ (P2).

It was stated that improvements to the national health system will not be possible until governance is strengthened at the national and district government levels to tackle ‘the fact that significant health moneys are lost to theft and corruption on areas such as supply of materials, drug procurement, contracts for construction of health facilities money’ (P8).

#### Drug supply

The availability of medical drugs and particularly drug stockouts were the issues raised by the greatest number of interviewees (P1, P4, P7, P9, P12, P13, P15, P16, P17, P20). Stockouts were attributed to limited government funding for drugs, a fragmented drug procurement system, inadequate drug supply and distribution, theft, and political disinterest in providing drugs and medical devices to specific vulnerable groups e.g. people with albinism, prisoners and LGBT communities. For example, ‘there is lack of resources to fund disease’ treatment for prisoners. Health of prisoners is not a priority’ (P7) and ‘there are always drug stockouts, yet we continue with the same inefficient supply chain’ (P9). When there are drug shortages, ‘the patients have to find money and buy medicines which they have been prescribed’ (P16).

The government’s drug policy is that they have ‘a final say on what to do with the drugs, where to distribute them and how to distribute’ (P16). Some drugs are included in the EHP, but additional drugs may need to be procured for conditions not covered (P4, P19). The government was perceived as not understanding the differences in the needs of different communities: ‘in reality, each district orders drugs based on the local needs and dynamics. The EHP in the HSSP II provides an ideal scenario, but in practice we have to respond to local realities’ (P19). The government was considered impervious to variation in population needs - even when evidence of a need for drugs/medical devices can be provided using information management systems, the government had failed to respond:

‘there is insufficient availability of lubricants and dental dams. There should be coordination to estimate the needs and purchase the sufficient amounts. [We have] statistics on the need of lubes and the state should use the supply-returns [the system for accounting for lubricants distributed] and the state can purchase based on the trends from it’ (P14).

The government requires that all national health system drug-procurement is via the Central Medical Stores (CMS) or their approval is sought before using other sources or distributing donations (P5). When this procedure is followed the supply can be poor, sometimes drugs are available in the CMS but not received by the hospitals (P13). There were calls from the interviewees for an improved system to coordinate between the CMS and the hospital pharmacies, and better auditing of drugs at healthcare facilities (P4, P13). In reality, drugs are accessed from a variety of sources i.e. the CMS, District Health Officers, donors and disease-specific programs (P5) and CHAM all use their preferred suppliers as an alternative to the CMS (P5, P15). For example, ‘since the city assembly is autonomous, they have at times decided to purchase from preferred local suppliers’ and ‘we have prequalified suppliers each and every year then we negotiate the prices and we buy our own drugs’ (P15). Further, when the service providers receive donations of drugs (including prenatal multivitamin tablets for pregnant mothers) from ‘international well-wishers’ they are supposed to consult the government about distribution but instead, they distribute them as they see fit, according to the needs of the community (P15). Other providers refuse them as ‘the drugs received are based on donor preferences. The MoH has given District Health Officers powers to refuse drugs which are not needed because it costs more to receive drugs that will not be used’ (P19).

This fragmented system of different facilities procuring drugs from different suppliers requires effective information management. One NGO was developing an information management system to harmonise the supply chain across their facilities by getting an ‘overall picture of supply of medicines (P1). They described the scale of the project:

‘to support harmonisation of the system including all drugs that run through the system, and donor-run supply chains e.g. USAID’s global Health Supply Chain. The government system ideally should take account of drugs that are procured by development partners [donors]. Ideally, a country should have one drug procurement agent but the current system in the country is chaotic’ (P1).

### Influence in decision-making

The health stakeholders interviewed consistently felt that they did not have any power to influence healthcare decision-making, particularly in the development of health policy (the NHP II and HSSP II); whilst donors were perceived as exerting a, largely positive, governing influence over the government.

#### Unequal power

Examples of unequal power and its impact in different health system contexts (governance bodies, services, donor-government relations) were identified.

A human rights organisation with a health-focus spoke of a lack of political will for greater governance as the reason why corruption and financial irregularities in construction contracts for healthcare facilities (which are centralised and led by the MoH) occurred, ‘government has enough authority over public services, but political will is crucial for things to work’ (P8). The deficit in health sector governance is exacerbated by a lack of top-down leadership:

‘the systems for effectiveness are simply not there in the public health sector and in the end there are chaos. The lack of functioning across the system is worsened by the fact that we do not have the leadership that understands the importance of functional systems and how much it would save on time and resources’ (P21).

They added that the governance mechanism of the parliamentary committee on health is underfunded, ‘meetings of the committee only takes place when parliament is able to fund the committee’. Governance is not considered a priority by the MoH and the Government of Malawi more broadly, ‘having a well-funded and functional committee is not a priority at the moment. Nothing will change in terms of legislative oversight without additional funding’ (P21).

Arguably, unequal power over the health sector is also maintained by the MoH’s insistence on oversight of top-level appointments to the boards/committees of organisations and facilities which receive (partial) government funding, such as the National AIDS Commission and Queen Elizabeth Central Hospital (P18, P17). For example, in the 2018 HIV and AIDs Act, the MoH retained the powers to approve high-level appointments (made by the board) in the National AIDS Commission, despite this public-private institution being established in law as independent of the government (P18). This degree of government oversight raises questions about transparency and the risk of corruption. There were also concerns about disproportionate influence in the relationship of the government to health facilities. For instance, when the Queen Elizabeth Central Hospital sought a change in status from government to private or public-private funding, the government appeared obstructive. The preferred option, to become a statutory corporation (a private-public concern with the government as the majority stakeholder), would have required a new law. It had the support of the public but not the politicians and no law was enacted, ‘the idea died because there was no political will to prepare the law and submit to parliament’ (P17). The issues of delayed payment to CHAM facilities were also considered political: ‘somehow when it is issues to do with the MoH the political part of it you can’t avoid’ (P4).

Donors were perceived as the only health stakeholder to exert any influence over the government and health system governance, possibly even requiring the development of a strategic framework for the NHP II as a condition of aid (i.e. the HSSP II) (P2). There was the widespread perception of greater governance when donors were involved (P8, P15, P18, P21), ‘the challenge in Malawi is that things only work when there is a donor funded project which has a higher standard of accountability in terms of milestones and reporting’ (P21). Since the collapse of the Sector wide Approach (SWAp; a donor-government partnership to map international funding to the activities of external development partners in Malawi), during which donors had close collaboration with the government, there has been less supervision of health funding (P15). Donors continue to fund the essential resources of the government’s healthcare facilities (e.g. electricity and water), but these are no longer paid into a basket fund shared with the government via the Ministry of Finance. There are much tighter controls on their use, with budget lines for specific organisations/programmes e.g. HIV-specific resources for the National AIDS Commission (P18). Due to a distrust of the government, some donors continue to operate in Malawi but independent of the government:

‘since cashgate, donors do not trust the government system and cannot transact their resources through the government system. So far USAID is not open to cooperating or collaborating with the government systems, but DFID is more open to collaboration or harmonisation’ (P1).

The internal governance mechanisms used by donors, international NGOs and multi-level organisations were regarded as indirectly affecting healthcare governance. For example, Oxfam conducts citizen satisfaction surveys to assess the impact of their programmes. These baseline and monitoring assessments are used to guide the programme’s strategy and assess its success but are also used to determine the focus and provide evidence for their advocacy agenda (P8). Ultimately, it was felt that the donors had and could have great influence over governance in the health sector, ‘donors have leverage because their funds are the lifeline of the health sector. So, everyone has to listen to their views’ (P8).

#### Stakeholder engagement

Stakeholders viewed the government as always putting their agenda ahead of the interests of the service providers and public (P4, P8, P17). Eleven interviewees (50%) had some involvement in either the development or validation of the NHP II and/or the HSSP II. Five (22.7%) felt this involvement was inadequate as not enough time was allowed for civil society or local government consultation, or that their involvement was a government afterthought (P6, P8, P17, P19, P20). As such, they felt that their priorities (the affordability, availability and accessibility of health services) were either not reflected in these documents or in insufficient detail (P6, P18). For example, the ‘NAC [National AIDS Commission] is mentioned in the NHP II and HSSP II, but only in passing and without much detail as to how the strategy or plan will manage HIV and AIDS and coordination around it’ (P18).

Efforts were made by the government to consult civil society and local government health stakeholders, with the significant involvement of MHEN (comprised of CSOs) (P2, P13). Despite this, CSOs that were not consulted reported that ‘the documents do not reflect the voices of patients’ (P13) and the local government representatives felt that they had minimal involvement (P19, P20):

‘involvement during the processes for developing the NHP II and HSSP II mostly happened at the MoH headquarters level. There were times when the District Health Office would be involved. Teams from the ministry headquarters would go to the districts with a questionnaire to ask questions related to policy and strategy. Consultations to finalise the two documents mostly happened at the Ministry headquarters’ (P19).

The NHP II and HSSP II were perceived as ‘this is a MoH thing. It’s also very political’ (P4), ‘[they] are only political tools which are mentioned in political speeches to show progress in the health system’ (P21). They were perceived as developed to appeal to the donors as it was donor-driven and funded (P1, P18, P21) but there was little confidence in the implementation of the policy and strategic plan (P4, P21), especially its ability to deliver the EHP (the starting point for the move towards UHC):

‘it is all about politics, but little to do with bringing change to better lives of the poor Malawian. Whatever the case, what Malawi is promising in the policy and strategy in terms of the essential health package, it cannot sustain due to domestic funding constraints’ (P21).

## Discussion

The interviews were conducted around 18 months after the introduction of the NHP II and HSSP II, the policy and framework for 2017–2022 which outline the strategies towards achieving UHC. We discuss our results and their implications for the key leadership and governance strategies: NHP II Priority Area 2 - Leadership and Governance; and HSSP II Objective 7 - Improve leadership and governance across the health sector and at all levels of the health system. Due to the limited literature on healthcare governance in Malawi (at any level - national, district, and service), our results can be viewed as providing both a baseline assessment and an early indication of progress toward implementing the policy. The following discussion is a reflection on these government documents with reference to our findings and other relevant literature, where available.

In the NHP II, the Government of Malawi identified serious challenges in leadership and governance at all levels which adversely affect service delivery and other health system functions: insufficient capacity (health resources, infrastructure and research); poor risk management; centralised decision-making; inadequate community empowerment (linked to answerability); and poor coordination and enforcement of policies and regulations. The government also identified inadequate communication and poor coordination with other health stakeholders (donors, international and national NGOs and networks, faith-based organisations, CSOs), resulting in poor alignment of these other stakeholders with national priorities, fragmented implementation and little harmonisation of plans and budgets, including parallel procurement.

Our findings, which emerged from semi-structured interviews which did not include specific questions about governance issues, mirror this list. This degree of consistency highlights the considerable awareness of both the government and health stakeholders of the challenges to service delivery and health system strengthening as well as the central importance of governance to health system efficiency and achieving UHC.

### Accountability and influence

There is widespread recognition that greater social accountability supports more responsive health policies and more effective services, and of the need for leadership to drive the strengthening of governance in the health sector [9, 41]. Social accountability means building answerability through the engagement and direct or indirect participation of citizens/the public. To work effectively, accountability requires openness, dialogue, enforceability (ensuring an action is taken and consequences or remedies for a failure to do so), honesty and responsiveness on the part of politicians, policy-makers, and providers to explain and justify their actions) [42].

In Malawi, the need for greater accountability comes from a demand for demonstrable results (improvements in health outcomes) and funding relationships i.e. where public money is being spent it must be accounted for [9]. Where funding is provided by external development partners (i.e. donors) effective governance is also demanded and implemented. In the HSSP II, the government admits that ‘there was mixed progress with respect to governance of the health sector over the past five years’ (the 2013–2017 period of the NHP I and HSSP I) (p18). To ensure progress over the following 5 years, the NHP II and HSSP II outline performance indicators and activities that can be monitored to achieve the necessary improvements in leadership and governance.

The NHP II includes a table of performance indicators (the monitoring and evaluation plan) to meet the objective of improving leadership and governance across the health sector. The baseline and measurable targets for each of the eight performance indicators are stated with requirements and assumptions/risks to achieving them. For example, by 2030 every central hospital will have achieved autonomy from the government. This requires a hospital board (verifiable via the annual Health Sector Reform Report), ‘(sustained) willingness to foster governance and accountability’, the availability of resources, and decentralisation. However, in our interviews, it was reported that efforts by Queen Elizabeth Central Hospital to seek greater autonomy had been obstructed by the government. In the NHP II and HSSP II, and reflected in the concerns of the interviewees, there is no guidance on how this culture of governance and accountability will be fostered and sustained or who will be responsible for monitoring the performance indicators, at what intervals and any sanctions available for enforcement.

It is possible that a lack of leadership and accountability from staff at the service level may be hampering achievement of the specific targets related to healthcare facilities [40]; however the interviewees were more concerned by the apparent lack of government-led leadership and oversight in the implementation of policies and felt limited in their ability to hold the government to account for their (in) actions. They identified tokenistic public engagement in government health decision-making, such as via the TWGs which are responsible for providing leadership and governance on health issues at the central level. A review of the functionality and effectiveness of the TWGs is specified in the HSSP II implementation plan (to achieve Objective 7) as an activity for the first 12 months (2017–18). The government acknowledges that ‘the activity and quality of guidance provided by TWGs has varied considerably with some meeting monthly while others have been inactive’ (p18). Our findings show that these TWGs sometimes include representatives from civil society and faith-based organisations but frequently stakeholder groups felt unrepresented or their contributions were undervalued. There was no indication from our data that this review had been completed, certainly none that the health stakeholders were involved, or that any recommendations had been implemented. Without a model of co-governance [43], whereby both top-down leadership drive and monitor the requirements and effective social accountability mechanisms to hold the government to account for their (in) actions, it is hard to see how the government can meet their performance indicators within the specified time period or at all.

The activities included in the 12 month implementation plan largely review the existing processes and develop guideline documents and monitoring infrastructure (e.g. to develop and implement an improved financial record keeping system and develop a Health Sector Aid Harmonization Manual). This focus on administrative and supportive systems may explain why our interviewees did not report improvements in governance or service delivery. The planned construction of new District Hospitals in five districts and 900 Health Posts is part of a longer term (5 year) plan (although none of the interviewees mentioned planned new facilities). Alternatively, the first year activities may not yet have been achieved and the associated guidelines disseminated. For example, a listed activity is ‘to develop a document explicitly outlining district governance structures providing clarity on roles, membership and linkages including developing and disseminating terms of reference for decentralised governance structures’ (p77). One interviewee explicitly stated that they had received no guidance on the role and composition of the health resource allocation committee mandated by the MoH to the district level. This may imply a delay in achieving or issues disseminating the outputs of the first 12 month plan. Communication between the government and health stakeholders is identified as both desirable and a challenge in the NHP II, but it appears that no meaningful progress has been made.

Concerns about the fragmentation of healthcare caused by a lack of coordination between government and donor policies and programmes are raised in the NHP II. This leads to duplication and inefficiency and makes it harder for the government to have oversight over the health system. The MoH established the Aid Coordination Unit in 2016 to minimise financing and activity duplication by ensuring alignment of health stakeholders to the HSSP II’s objectives and strategies. Based on our interviews, the situation does not appear to have improved since 2016. The interviewees indicated a desire for greater communication with the government but a deep mistrust of their ability to generate the conditions for greater collaboration. They doubted the government’s willingness (and sometimes ability due to limited resources) to meaningfully consult and implement the recommendations of health stakeholders (via adequate answerability processes) [41]. The government states in the HSSP II, that there are some local oversight institutions, with stakeholder coordination mechanisms established by the District Health Offices but that these governance bodies are not very functional. In the absence of adequate government-led governance, service-providers and CSOs together with NGOs and donors are implementing their own accountability mechanisms; although the interviewees felt that only donors had true influence with the government, even insisting on the development of the HSSP II as a precondition to aid. However, even then, doubts were raised by the interviewees and in the literature over the government’s willingness to improve governance sufficiently to regain donor confidence and work collaboratively [10, 44].

### Resource management

The HSSP II attempts to plan more realistically than the first iteration of the HSSP, but ‘there are still large resource gaps and optimistic targets in all areas of the health system’ (p58). This seems to serve as a disclaimer for not achieving the overall objectives and specific targets within the 2017–2022 timeframe. An overarching issue faced by the government is the need for additional funds to improve governance (e.g. to pay for information management systems, to hold regular meetings of governance bodies and to ensure timely payment of service-providers according to the SLAs).

The HSSP II includes a costing for the activities required to deliver the EHP together with the resources to strengthen the health system (including health resources, health information systems and governance). The activities required to achieve Objective 7 require 2,220,287 USD in the first year (2017/18), decreasing each subsequent year. This equates to 0% of the total 5 year budget, and the MoH notes that the budget continues to outstrip the resources. The fiscal deficit inhibits greater governance, whilst poor governance accentuates the deficit. For example, through allowing leakage in the drug supply, understaffing in health facilities, and unnecessary duplication of management and services at national and local levels. The government hopes that improved governance will improve the fiscal space (the budgetary room available to a government to provide resources for public needs without affecting fiscal sustainability) [45]. It is our concern that where cost savings must be made, the governance activities will continue to be inadequate, as already appears to be the case 18 months after the introduction of the HSSP II.

The government has long recognised the need for improved financial management, but their ability to enact the Financial Management Improvement Plan (FMIP) formulated in 2012 was itself hampered by insufficient resources - the limited number of skilled staff and information technology equipment affected the capacity of the MoH finance department. The Internal Audit Unit was created in 2008 to improve the accountability of public resources and reduce the risk of corruption and financial mismanagement in the government-funded health system [46]. However, the government reports that the Internal Audit Unit for the MoH was unable to conduct visits to health facilities due to insufficient funding which limited staff and equipment capacity, preventing it from performing its governance function. Insufficient funding also prevents the procurement plans produced each year from being implemented, resulting in ad hoc procurement and the accumulation of arrears. This is a probable cause of the fragmented procurement in the health system reported by the interviewees, whereby local government and service-providers seek assistance from their own preferred suppliers and donors as they cannot rely on the government’s procurement and supply systems.

The HSSP II outlines how the health system will be strengthened to support the delivery of the EHP (in the move towards UHC by 2030). A key strategy is to prioritise filling staff vacancies that deliver the EHP [47]. The interviewees were particularly concerned that the concentrated focus on the EHP risks increasing rather than decreasing inequalities in health as some groups would be further marginalised (e.g. LGBT populations). The government acknowledges that difficult decisions regarding the conditions to prioritise for the EHP were made and continue to be as:

‘despite the cost of the revised EHP [outlined in the HSSP II] being closer to the resources available for its provision than before, the cost continues to outstrip resources. The result of this is that, even assuming no health system constraints, it will not be possible to deliver the new EHP to the entire population in need. It is important that ongoing discourse around the EHP focuses on the budgets available for its provision’ [47].

This stance makes it unlikely that government-funded bodies (other than the NAC) will be able to access top-up funding or that the government will be able to plug gaps in the service provision and drug availability at the district and city assembly levels, both issues identified by the interviewees.

We note that the coronavirus pandemic has occurred since the publication of this document. The Malawian government response has to balance the risk of adopting a ‘lockdown’ policy prioritising the protection of health and the health system versus the economy, particularly considering the potentially severe impact of closing essential facilities (e.g. market places) on people already living in poverty [48]. Without considerable external assistance and collaboration between health stakeholders and possibly an intersectoral approach, the EHP (let alone UHC) will be undeliverable and existing inequalities in health and healthcare access will rise (especially if user fees are introduced) [19, 49–51].

### Limitations

A range of stakeholders from different organisation types were recruited to the study, but there were more representatives from civil society than the other organisation types. All stakeholder perceptions were reported regardless of the number of participants to comment on each issue, issues of greater importance for CSOs may have been over-reported. Interviews were conducted with stakeholders based in Lilongwe and Blantyre but drew on their experience of both urban and rural healthcare in these districts, and from projects in other districts such as Karonga and Salima. As the stakeholders chose to be interviewed in their places of work, the location was not perceived to constrain, and thus potentially bias, the conversation, especially as anonymity had been assured. The need to protect the identities of the interviewees limited the transferability [52]. The semi-structured interview approach, with a focus on open-ended questions, increased the breadth of the data collected but reduced the repeatability of the study.

The interviews were recorded via field notes and transcripts which were not participant-verified. Although guidelines for making field notes were followed, it was impossible to completely mitigate the risk of interviewer subjectivity in the recording and transcription of the field notes [52]. The interviewer was considered largely free of preconceptions about the health system and healthcare governance as they had not previously worked in the health sector and had lived outside Malawi for some time. Instead, their Malawian nationality and health sector ‘outsider’ status may have enabled the interviewees to express their opinions more openly. There were no concerns that the conversations were biased by the apparent knowledge of some of the interviewees of the Thanzi la Onse project, of which this study is part [45].

The governance issues identified by the participants, and examples given, may pre-date the NHP II and HSSP II. However, as the interviewees also identified current and ongoing efforts to address the challenges, the governance issues were considered outstanding at the point of the interview (unless otherwise stated).

## Conclusions

This exploratory study captured a diverse range of stakeholder perspectives on health system functioning, and through the lens of healthcare governance in Malawi, gives an overview of challenges to the achievement of UHC in low income countries in this critical decade running up to 2030. Our findings suggest that the Government of Malawi, via the NHP II and HSSP II, is making policy and strategic efforts to improve governance in the health system at all levels. However, 18 months after the publication of these documents, health sector stakeholders have seen little improvement in key areas of governance: accountability, stakeholder engagement in decision-making, and health resources. Our findings highlight ongoing challenges to the government’s aim to improve healthcare governance and strengthen the health system which, without greater leadership and investment in governance mechanisms (including policy monitoring and enforcement), are preventing delivery of the EHP and will prevent achievement of UHC. Involving stakeholders, even when their views are uncomfortable for governments and highlight governance failures, will lead to better services.

## Supplementary Information

**Additional file 1.** Interview questions.

## Data Availability

The datasets generated and analysed during the current study are not publicly available as analysis is ongoing for additional publications but are available from the corresponding author on reasonable request.

## Abbreviations

CMS: Central Medical Stores
CHAM: Christian Health Association of Malawi
EHP: Essential Health Package
LGBT: Lesbian, Gay, Bisexual, and Transgender
MHEN: Malawi Health Equity Network
MoH: Ministry of Health
NAC: National AIDS Commission
NGO: Non-government organisation
NHP: National Health Policy
HIV: Human immunodeficiency virus
HSSP: Health Sector Strategic Plan
SLA: Service level agreement
SWAp: Sector wide Approach
TWG: Technical working group
UHT: Universal Health Coverage
