# Supplementary material for: Challenges to effective governance in a low income healthcare system: a qualitative study of stakeholder perceptions in Malawi
Source: BMC Health Serv Res. 2020 Dec 14;20:1142. doi: 10.1186/s12913-020-06002-x (PMC7734892; doi:10.1186/s12913-020-06002-x)
Supplement: Supplementary file 1 — Additional file 1. Interview questions. [file 12913_2020_6002_MOESM1_ESM.docx]

Supplementary material 1 – Interview questions

The following guiding questions were used by the interviewer to prompt the interviewees to elaborate on their and their organisation’s experience and perception of the functioning of the health system and healthcare decision-making in Malawi. Context and conversation-specific questions were then asked responsively e.g. how does your organisation coordinate with the District Health Office?

1. Please tell me about the (health-related) work of your organisation.
2. What challenges do you see in Malawi’s health sector?
3. What are the challenges to decision making in the allocation of health care resources and health decision-making?
4. What interaction do you have with other areas of the health sector e.g. health facilities, central or district health institutions, health-related CSOs?
5. Do you collaborate with the government by influencing health policy and/or budgeting?
   1. If yes, to what extent were you and your organisation involved in the development of the national health policy and health sector strategic plan?
   2. If yes, to what extent were you and your organisation involved in the development of the health budget?
6. What are the priority issues for your organisation and how can they be addressed/advocated for at the government level?
7. Are your priorities influenced by the HSSP or do you identify them on your own?
8. Do you think there is an opportunity for you at your level to get involved in health policy-making?
9. How do you see the role of donors and do you work with donors on some matters?
   1. If yes, do you receive any funding from donors, and what are the mechanisms for decision making with them?
10. How does your organisation organise resource allocation decision making and budgeting and what are the challenges?
